# A Link No Longer Missing: New Evidence for the Cetotheriid Affinities of *Caperea*

**DOI:** 10.1371/journal.pone.0164059

**Published:** 2016-10-06

**Authors:** Felix G. Marx, R. Ewan Fordyce

**Affiliations:** 1 School of Biological Sciences, Monash University, 25 Rainforest Walk, Clayton, Victoria, 3800, Australia; 2 Geosciences, Museum Victoria, Melbourne, Australia; 3 Directorate of Earth and History of Life, Royal Belgian Institute of Natural Sciences, Brussels, Belgium; 4 Department of Geology, University of Otago, Dunedin, New Zealand; 5 Departments of Vertebrate Zoology and Paleobiology, National Museum of Natural History, Smithsonian Institution, Washington DC, United States of America; Virginia Commonwealth University, UNITED STATES

## Abstract

The origins of the enigmatic pygmy right whale *Caperea marginata*, the only living member of its subfamily (Neobalaeninae), are an outstanding mystery of cetacean evolution. Its strikingly disparate morphology sets *Caperea* apart from all other whales, and has turned it into a wildcard taxon that holds the key to understanding modern baleen whale diversity. Morphological cladistics generally ally this species with right whales, whereas molecular analyses consistently cluster it with rorquals and grey whales (Balaenopteroidea). A recent study potentially resolved this conflict by proposing that *Caperea* belongs with the otherwise extinct Cetotheriidae, but has been strongly criticised on morphological grounds. Evidence from the neobalaenine fossil record could potentially give direct insights into morphological transitions, but is currently limited to just a single species: the Late Miocene *Miocaperea pulchra*, from Peru. We show that *Miocaperea* has a highly unusual morphology of the auditory region, resulting from a–presumably feeding-related–strengthening of the articulation of the hyoid apparatus with the skull. This distinctive arrangement is otherwise only found in the extinct Cetotheriidae, which makes *Miocaperea* a “missing link” that demonstrates the origin of pygmy right whales from cetotheriids, and confirms the latter’s resurrection from the dead.

## Introduction

*Caperea marginata* is the most enigmatic and disparate of all living baleen whales (Mysticeti) in terms of its morphology, behaviour and even sensory abilities [1–4]. The evolutionary origins of *Caperea* remain highly controversial, which, given the status of this species as the sole representative of one of the four extant mysticete (sub)families, represents the greatest obstacle to understanding the true scope and structure of modern baleen whale biodiversity. Morphological cladistics generally ally *Caperea* with right whales [5–7], whereas molecular analyses consistently cluster it with rorquals and grey whales (Balaenopteroidea) [8–10]. A recent study [4, 10] potentially resolved this conflict by proposing that pygmy right whales belong with the otherwise extinct Cetotheriidae, but has been criticised for allegedly not considering ontogenetic change, and for making wrong assumptions about character homology [6, 11, 12]. Fossil neobalaenines could potentially give direct insights into morphological transitions, but the only available material–*Miocaperea*–is phenetically close to *Caperea* [13] and, consequently, has so far remained largely uninformative in this regard. Here, we re-examine *Miocaperea* with a particular focus on the phylogenetically informative ear region, and show that it shares with cetotheriids a previously overlooked, yet distinctive and highly unusual auditory morphology to the exclusion of all other mysticetes (including *Caperea*).

## Material and Methods

The re-description of the auditory region of *Miocaperea pulchra* is based on the holotype and only known specimen, permanently housed at the Staatliches Museum für Naturkunde Stuttgart, Germany (specimen number 46978). No permits were required for the described study. The phylogenetic analysis uses the total evidence matrix of Marx and Fordyce [10], with all subsequent additions and corrections [14, 15]. As a result of our new observations on *Miocaperea*, we amended our data by: (i) rewording and recoding characters 127 (“Lateral lamina of pterygoid”), 162 (“Anteromedial corner of pars cochlearis in ventral view”), 182 (“Facial sulcus on compound posterior process”) and 183 (“Position of facial sulcus on compound posterior process in ventral view”); (ii) ordering Char. 182; and (iii) adjusting the scorings of chars 157 (“Position of lateral tuberosity”) and 198 (“Dorsomedial corner of sigmoid process in anterior view”).

The cladistic matrix including all new and amended scorings is available as Supplementary Material (S1 File), and from MorphoBank (http://www.morphobank.org/), project 2331: the full matrix is stored in the “Documents” section. The analysis was carried out without any clock assumptions in MrBayes 3.2.6 [16], on the Cyberinfrastructure for Phylogenetic Research (CIPRES) Science Gateway [17] (20 million generations, first 25% of generations discarded as burn-in). To determine the distribution of an anteriorly expanded paroccipital concavity, we traced the phylogenetic history of Char. 182 using both parsimony-based and likelihood-based ancestral state reconstruction methods in Mesquite 3.04 [18]. Because we are primarily interested in the presence of the paroccipital cavity and attendant posteroventral flange on the posterior process, we treated states 1 (posteroventral flange present) and 2 (posteroventral flange present and markedly enlarged) as the same for the purpose of state reconstruction.

## Results and Discussion

### Re-description of the auditory region of *Miocaperea pulchra*

**Fig 1:**
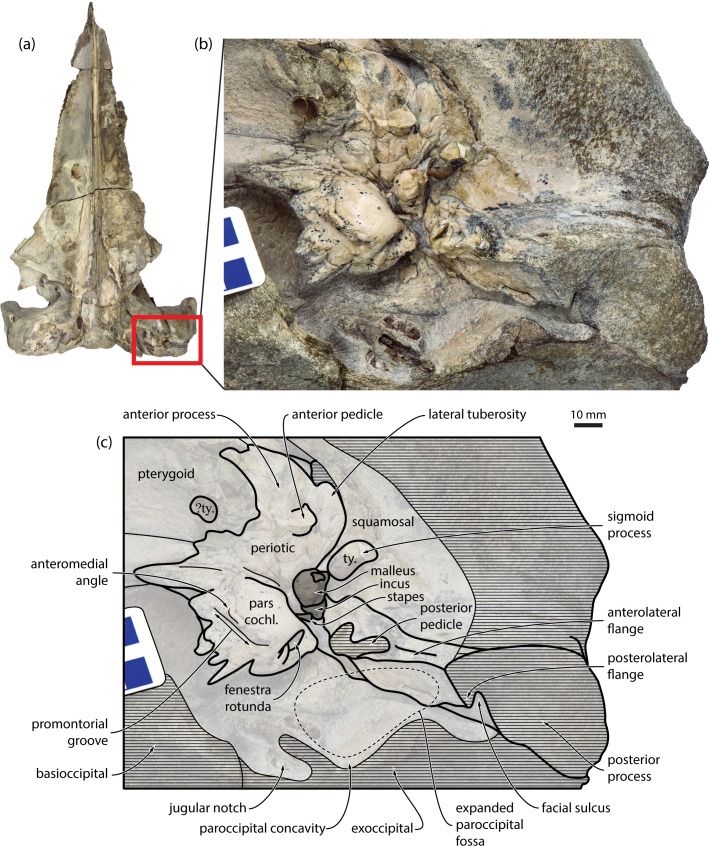
Detailed morphology of the auditory region of *Miocaperea pulchra* (SMNS 46978). (*a*) skull in ventral view, with the auditory region highlighted in red; (*b*) photograph and (*c*) line drawing of the auditory region in ventral view. pars. cochl., pars cochlearis; ty., tympanic bulla.

Both the left and right periotics of SMNS 46978 are preserved *in situ*, but the right is heavily eroded and broken. The following description will therefore be based on the left periotic (Fig 1), unless stated. Unlike in *Caperea*, the anterior process is firmly attached to the body of the periotic. In ventral view, the anterior process is relatively wide transversely and about as long anteroposteriorly as the pars cochlearis. There is a well-developed lateral tuberosity, which extends anteriorly just beyond the level of the anterior pedicle of the tympanic bulla. The shape of the lateral tuberosity is slightly obscured, but it appears to be blunt and relatively robust. In medial view, the dorsal portion of the anterior process is anteroposteriorly narrow and notably rises towards the cranial hiatus. Anteromedially, the anterior process is broadly underlain by the lateral lamina of the pterygoid. Because of this, the shape of the anterior border of the periotic can only be surmised, but appears to be markedly concave. Anterior to the pars cochlearis, the medial surface of the anterior process is somewhat concave, presumably marking the origin of the tensor tympani muscle; however, there is no associated ridge or shelf. The mallear fossa is mostly obscured by sediment, but its anteriormost portion at least appears to be poorly defined and shallow.

In ventral view, the pars cochlearis is bulbous, with a slightly angular, dorsally displaced anteromedial corner. Unlike in balaenopterids, the rim of the fenestra rotunda is flush with the posterior border of the pars cochlearis. The caudal tympanic process is short and oriented posteriorly. The fenestra ovalis, distal opening of the facial canal and fossa for the stapedius muscle are mostly or entirely obscured by sediment and/or the auditory ossicles (see below). In medial view, the anteriormost portion of the pars cochlearis is confluent with the dorsal border of the anterior process and thus somewhat rises towards the cranial hiatus. The rim of the internal acoustic meatus consists of a series of spike-like, cranially oriented projections, thus giving the dorsal margin of the pars cochlearis a jagged appearance. Ventrally, these spikes are offset from the remainder of the pars cochlearis by a step-like promontorial groove. The fenestra rotunda is relatively large and extends from the ventral border of the pars cochlearis almost to the level of the promontorial groove. Dorsally, the rim of the fenestra rotunda is interrupted by two sulci rising towards the dorsal surface of the pars cochlearis. Unlike in grey whales, the fenestra rotunda is not confluent with the aperture of the cochlear aqueduct. The caudal tympanic process is a small, rounded and somewhat posterodorsally directed plate, and is confluent with a small shelf forming the lateral border of the fenestra rotunda. As far as can be told, the caudal tympanic process is clearly separated from the crista parotica.

In ventral view, the distal end of the compound posterior process of the tympanoperiotic is markedly expanded both anteroposteriorly and dorsoventrally, and widely exposed on the lateral skull wall. The distalmost portions of both posterior processes are eroded. The broken base of the posterior pedicle of the bulla is robust and U-shaped, reflecting the internal excavation of the pedicle by the tympanic cavity. Laterally, the posterior pedicle is continuous with a transversely oriented anteroventral flange (*sensu* [14]). Posteriorly, this flange is bordered by a second, posteriorly oriented posteroventral flange (*sensu* [14]), which almost completely floors the facial sulcus. Together, the anteroventral and posteroventral flanges delimit a fossa on the ventral surface of the posterior process that is aligned with, and therefore forms part of, the paroccipital concavity. On the right, the paroccipital concavity has been largely obliterated by erosion, but the facial sulcus is preserved and ventrally floored, in the same manner and position as on the left (S1 Fig). Note that the paroccipital concavity was previously misidentified as the facial sulcus (p. 891 and Fig 13D in [19]). The posterior border of the paroccipital concavity is eroded, but the excavation of the exoccipital is clearly more pronounced than in *Caperea*. The external acoustic meatus has been largely obliterated by erosion.

**Fig 2:**
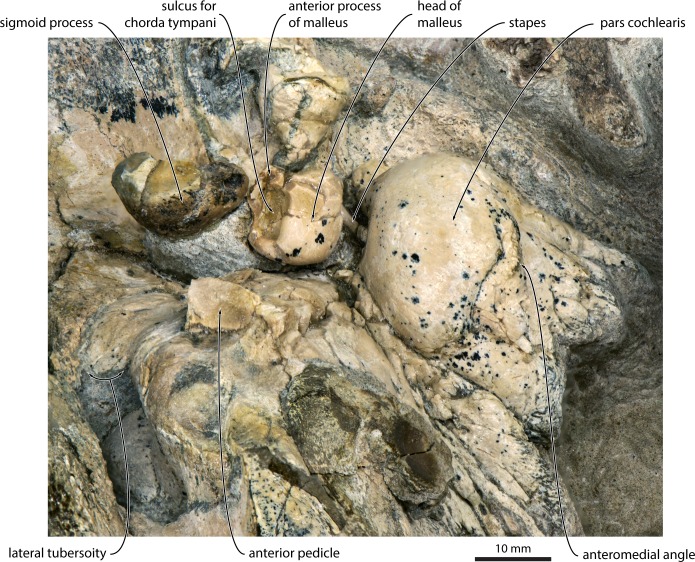
Detailed morphology of the auditory region of *Miocaperea pulchra* (SMNS 46978), in anterior view. Note the position of the dorsal portion of the sigmoid process adjacent to the head of the malleus.

Contrary to what is stated in the original description [19], the holotype of *Miocaperea pulchra* preserves both stapes, both incudes, the left malleus and the dorsal portion of the sigmoid process of the left tympanic bulla. On both sides of the skull, the auditory ossicles are naturally articulated and virtually *in situ* (Figs 1 and 2). The head of the malleus is situated immediately beside the dorsomedial apex of the sigmoid process in exactly the position as seen in other mysticetes, without, however, being fused to it as in balaenopterids (Fig 2). The articular facets for the incus are obscured; nevertheless, judging from the shape of the head, the vertical facet appears to be considerably larger than the horizontal one. As in *Caperea*, the tubercule is transversely short and stocky. The anterior process of the malleus is missing, but the anteroventral side of the head shows a large excavation corresponding to the dorsalmost portion of the sulcus for the chorda tympani. The incus is robust, with a well-developed body and crus longum. The lenticular process is mostly hidden from view, but can be surmised to be relatively large, based on both the shape of the crus longum and the head of the stapes. The crus breve of the incus and most of the stapes remain covered by matrix.

### Phylogenetic implications

**Fig 3:**
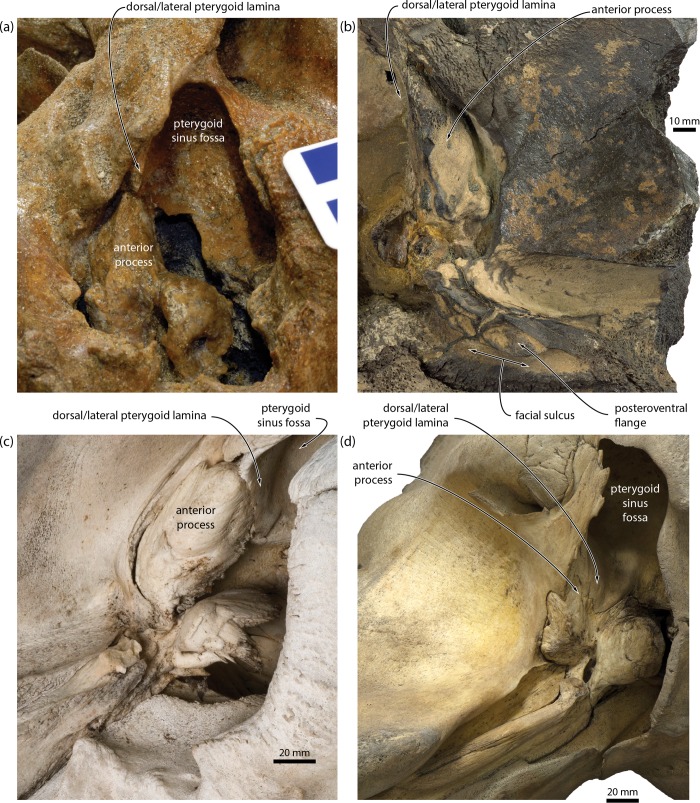
Extension of the lateral lamina of the pterygoid on to the anterior process of the periotic. (*a*) *Piscobalaena nana*, MNHN SAS1617; (*b*) *Herentalia nigra*, ZMA 5069; (*c*), *Caperea marginata*, OM VT227; (*d*), *Balaenoptera acutorostrata*, NMNS M42450.

We identified several new features of the auditory region that–contrary to the traditional morphological interpretation of relationships–ally *Miocaperea* with *Caperea*, cetotheriids and, in some cases, balaenopteroids, but not balaenids. Specifically, *Miocaperea* shares with (i) *Caperea* and the cetotheriid *Herpetocetus* the presence of an angled, medially projecting anteromedial corner of the pars cochlearis (Fig 1) [4]; with (ii) *Caperea*, *Herpetocetus* and certain (stem) balaenopteroids a lateral tuberosity of the periotic that extends anteriorly past the level of the anterior pedicle of the tympanic bulla [4]; with (iii) *Caperea*, the cetotheriids *Herentalia*, *Metopocetus* and *Piscobalaena*, and some balaenopteroids the extension of the lateral lamina of the pterygoid on to the anterior process of the periotic (Figs 1 and 3); and with (iv) cetotheriids, but not *Caperea*, the presence of an enlarged paroccipital concavity on both the anteroventral surface of the exoccipital *and* the posteroventral surface of the compound posterior process of the tympanoperiotic (hereafter, “posterior process”). The enlargement of the paroccipital concavity–previously misidentified as the facial sulcus [19]–is particularly striking, and accompanied by the development of a posteroventral flange (*sensu* [14]) flooring the facial sulcus (Fig 1).

**Fig 4:**
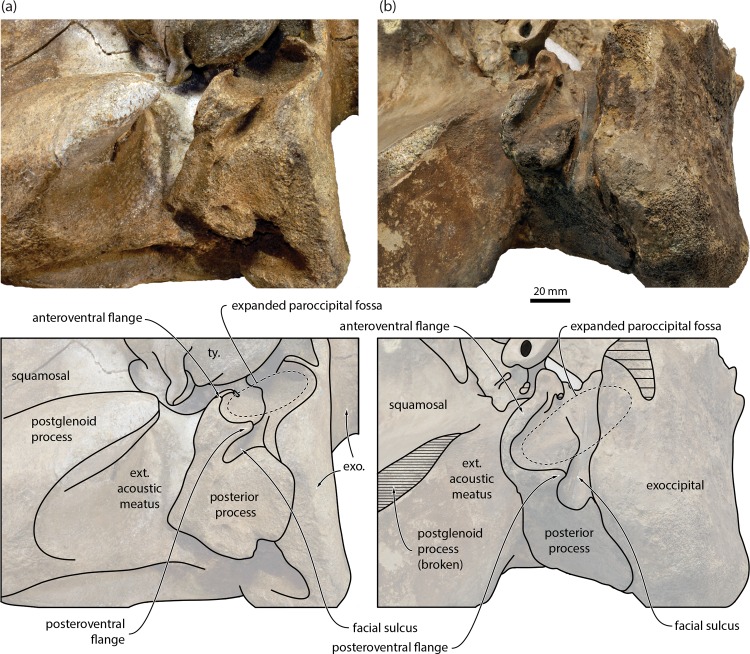
Auditory region of two representative cetotheriids in ventrolateral view. (*a*) *Piscobalaena nana*, MNHN SAS1616; (*b*) *Metopocetus durinasus*, USNM 8518. The photograph of *P*. *nana* has been mirrored to facilitate comparisons. ext. acoustic meatus, external acoustic meatus; exo., exoccipital; ty., tympanic bulla.

**Fig 5:**
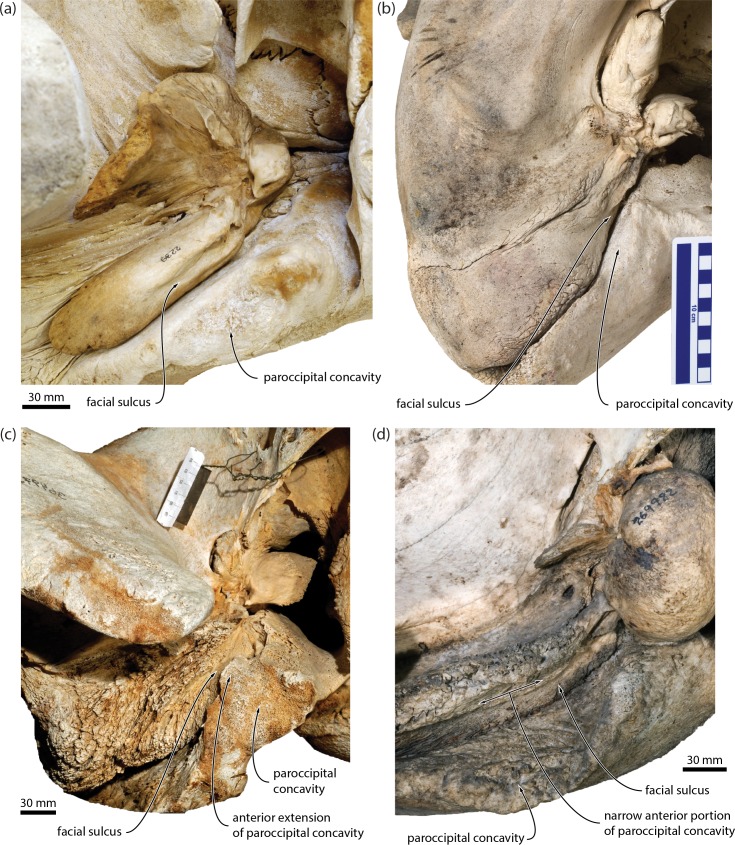
Auditory region of extant mysticetes. (*a*) *Eubalaena australis*, NMNZ MM002239; (*b*) *Caperea marginata*, OM VT227; (*c*) *Eschrichtius robustus*, USNM 364973 (mirrored to facilitate comparisons); (*d*) *Megaptera novaeangliae*, USNM269982. Note the enlarged paroccipital concavity in *E*. *robustus*.

The anterior expansion of the paroccipital concavity on to the posterior process is a highly distinctive feature, yet has been mentioned only twice in the cetacean literature, namely, for the cetotheriids *Piscobalaena nana* [20] and *Metopocetus hunteri* [14]. The same structure, including an attendant posteroventral flange, occurs in other species of *Metopocetus* (previously misidentified as either the external acoustic meatus [21] or the facial sulcus [22]), as well as *Herentalia*, “*Cetotherium*” *megalophysum* and *“Metopocetus” vandelli* (Figs 3 and 4). A less developed version occurs in *Brandtocetus*, *Kurdalagonus* and *Herpetocetus*. In the latter, a marked anterior shift of the facial sulcus has led to a corresponding reduction in the size of the posteroventral flange. Nevertheless, in some species (e.g. *H*. *morrowi*; UCMP 124950; SDNHM 34155) the flange is still developed well enough to close the facial sulcus in ventral view. No other extinct or extant mysticetes we examined show evidence of a posteroventral flange. An anteriorly extended paroccipital concavity does occur in some balaenopterids, e.g. certain specimens of *Megaptera novaeangliae*, but it is generally narrow and does not floor the facial sulcus (Fig 5). Similarly, the paroccipital concavity is enlarged in the extant grey whale (*Eschrichtius robustus*) [14] but, in lieu of a posteroventral flange, roofed by an anterior extension of the ventral surface of the exoccipital (Fig 5).

**Fig 6:**
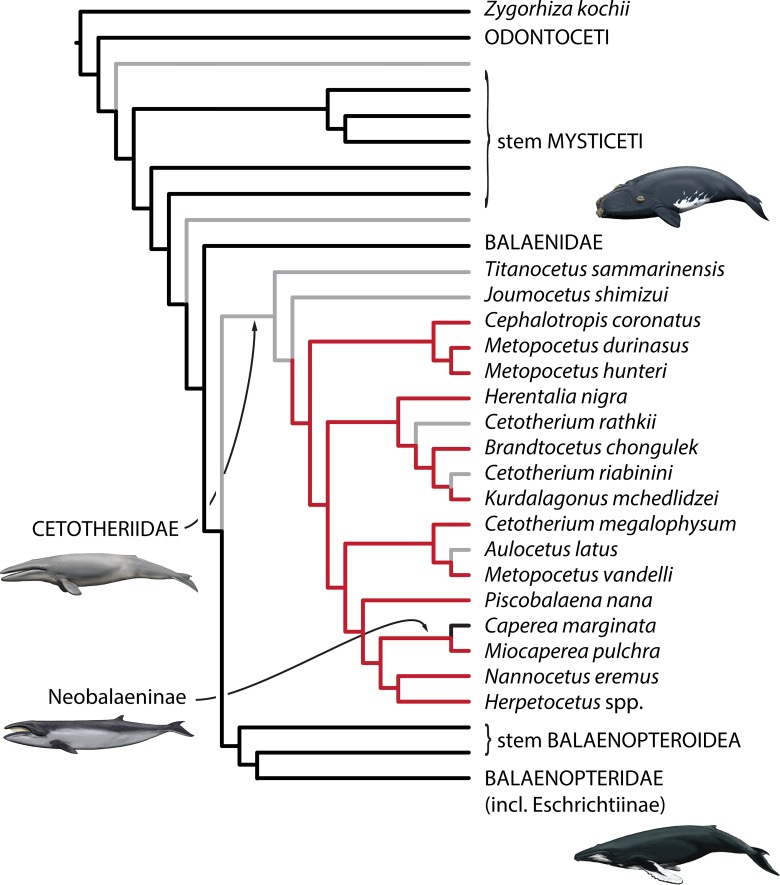
Phylogeny of mysticetes showing the occurrence of an enlarged paroccipital concavity. Summary of the results of the total evidence (non-clock) analysis. Note the nesting of Neobalaeninae within Cetotheriidae. Red and black branches denote the presence and absence, respectively, of an anteriorly expanded paroccipital concavity with an attendant posteroventral flange. Grey denotes missing data and ambiguous ancestral state reconstructions. Note the difference between *Caperea* and *Miocaperea*, with the latter matching other cetotheriids. Reconstructed states are based on parsimony, with a likelihood-based reconstruction yielding nearly identical results. Full results are provided in the Supplementary Material (S2 Fig).

The distinctively enlarged paroccipital concavity and associated posteroventral flange represent a previously unrecognised, taxonomically restricted feature of cetotheriids and neobalaenines, later lost again in *Caperea*. The presence of this structure in *Miocaperea pulchra* makes this species a rare ‘missing link’ uniting features otherwise unequivocally associated with cetotheriids and *Caperea*, respectively, and thus strongly supports an evolutionary relationship between the two (see below). This interpretation is borne out by our phylogenetic analysis, which groups all cetotheriids and neobalaenines into a monophyletic clade and reconstructs the expansion of the concavity on to the posterior process as a shared feature (Fig 6).

### Functional implications

Across Cetacea, the paroccipital concavity has long been interpreted as an osteological correlate of either the posterior sinus and/or the secondary, ligamentous articulation of the stylohyal with the basicranium [14, 23, 24]. A posterior sinus is generally thought to be present in mysticetes [24], but its existence is poorly established and inferred primarily based on (i) its occurrence in odontocetes, where it occupies some or all or the paroccipital concavity; (ii) the consistent presence of the paroccipital concavity in all mysticetes; and (iii) a study by Beauregard [25], which described the presence of a small “posterior” sinus in *Balaenoptera acutorostrata*. As far as we can tell, all other referrals to a mysticete posterior sinus in the literature are ultimately based on these points, with little or no direct data to confirm the occurrence of this structure in the living species.

**Fig 7:**
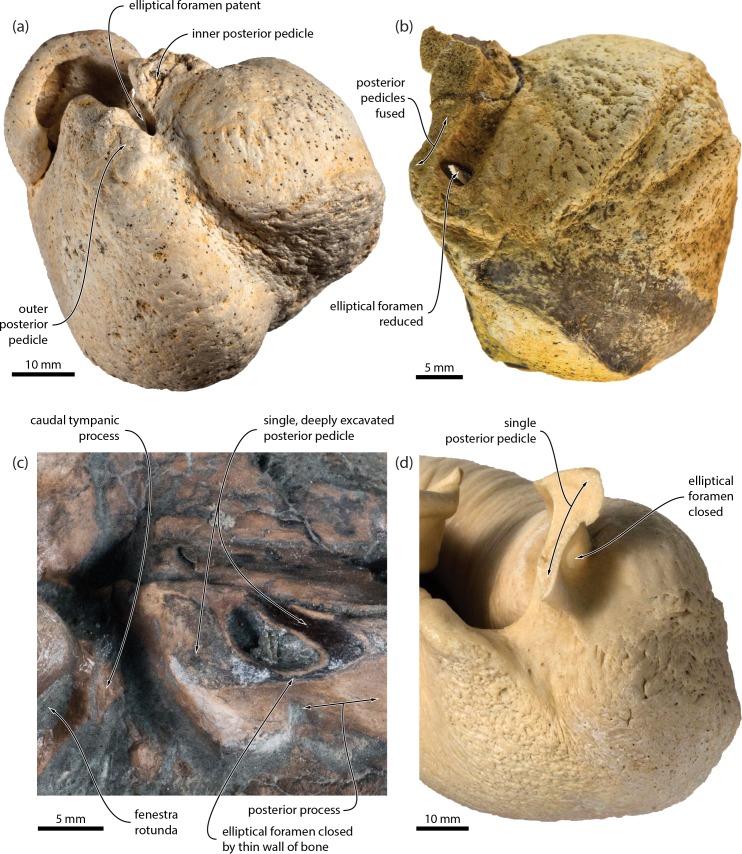
Posterior pedicle of the tympanic bulla and elliptical foramen. (*a*) posterior view of tympanic bulla of *Tokarahia kauaeroa*, OU 22235 (mirrored to facilitate comparisons), Late Oligocene; (*b*) posterior view of tympanic bulla of an undescribed chaeomysticete, OU 22732, Late Oligocene; (*c*) ventral view of broken posterior pedicle of an undescribed chaeomysticete, OU 22705, Early Miocene; (*d*) posterolateral view of tympanic bulla of *Balaenoptera bonaerensis*, OM VT3057; extant. Note the progressive closure of the elliptical foramen and the thin, translucent central portion of the posterior pedicle in *B*. *bonaerensis*.

In odontocetes, the posterior sinus emerges from the tympanic cavity via the elliptical foramen, which in turns separates the inner and outer posterior pedicles of the tympanic bulla [24]. Inner and outer pedicles are also present in archaic mysticetes, including all of the toothed species and eomysticetids, but are absent in all extant taxa. Previous interpretations of this situation implied the loss of the outer posterior pedicle [5], which would leave the posterior sinus wrapped around the remaining pedicle to extend, as in odontocetes, into the paroccipital concavity. However, new fossil mysticetes from New Zealand (OU 22705, 22732) now show that the outer posterior pedicle was not lost (Fig 7). Instead, the elliptical foramen was gradually closed along the lineage leading to crown mysticetes, as shown by a transformation series ranging from taxa with a well-developed elliptical foramen (e.g. the eomysticetid *Tokarahia kauaeroa*, OU 22235), to taxa with a partially closed, circular foramen (OU 22732), to taxa with a single posterior pedicle that is deeply excavated anteriorly and extends far onto the dorsal surface of both the involucrum and the outer lip of the tympanic bulla (OU 22705). In modern mysticetes, the remnant of this excavation can still generally be seen on the inside of the posterior pedicle, where it appears as a relatively small, dorsally directed lobe of the tympanic cavity. In some specimens (e.g. OM VT3075, a juvenile *Balaenoptera bonaerensis*), the bony wall behind this lobe is extremely thin and translucent (Fig 7), which likely marks the ancestral position of the formerly open elliptical foramen.

A second line of evidence for the closure of the elliptical foramen comes from the position of the tympanic sulcus, which marks the attachment of the tympanic membrane. In the bulla of archaic mysticetes with an elliptical foramen, the tympanic sulcus runs from the posterior surface of the sigmoid process on to the inside of the conical process, thence rising on to the inside of the outer posterior pedicle. The sulcus in living mysticetes essentially follows the same course, and posteriorly rises up on the inside of the single remaining pedicle. We regard the path of the tympanic sulcus as a phylogenetically and functionally conservative indicator of the outer posterior pedicle. At the same time, however, the single pedicle of extant mysticetes closely resembles the inner posterior pedicle of more archaic species in both size and position, thus suggesting that the two pedicles fused, as opposed to one of them being lost. If the elliptical foramen in crown mysticetes is closed, then it follows that the posterior sinus, which ancestrally exited through it, must have either disappeared or dramatically changed its course. Given the gradual reduction of the internal excavation of the posterior pedicle, we propose that the posterior sinus is absent in crown mysticetes, and that the small sinus described by Beauregard [25] probably was a small diverticulum of the peribullary sinus. This appears to be confirmed by the recent dissection of a minke whale, *Balaenoptera acutorostrata* (USNM 593594), by REF, which did not reveal any evidence of a posterior sinus.

**Fig 8:**
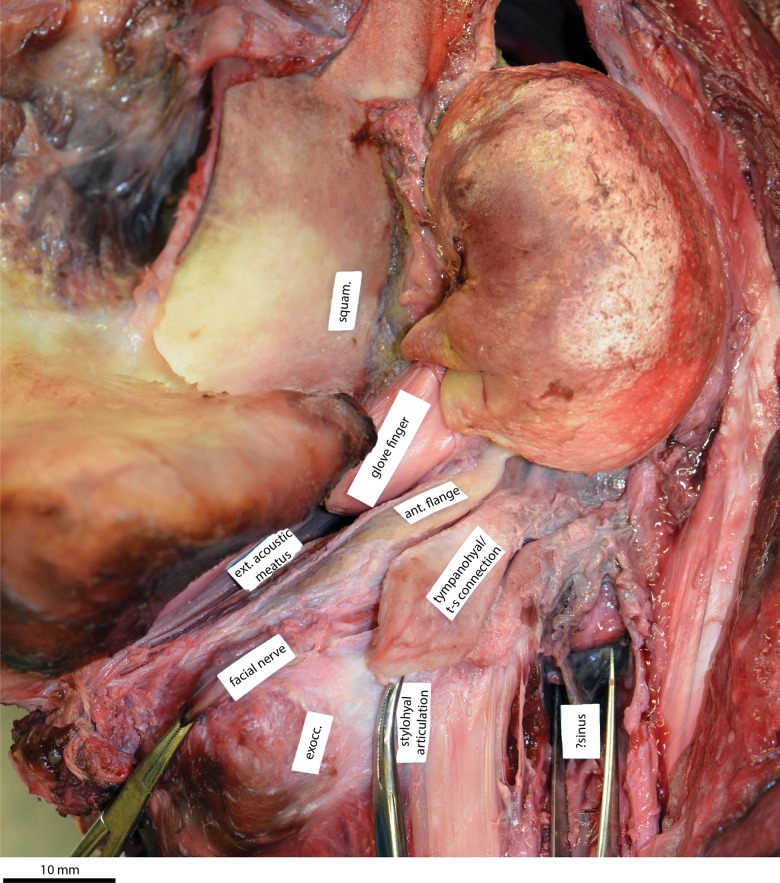
Dissected auditory region of a minke whale, *Balaenoptera acutorostrata*. Ventral view of the right basicranium of specimen USNM 593594, with periotic and bulla *in situ*. Note the robust cartilaginous tympanohyal, or connection between the tympanohyal and stylohyal. ant. flange, anteroventral flange; exocc., exoccipital; ext. acoustic meatus, external acoustic meatus; squam., squamosal.

In contrast to the posterior sinus, the association of the paroccipital concavity with the stylohyal is supported by the same recent dissection, which showed that the anteroventral flange of the posterior process houses a robust cartilaginous structure equivalent to either a portion of the tympanohyal or the connection between the tympanohyal and the stylohyal (Fig 8). We therefore propose that the size and/or shape of the paroccipital concavity may be related to feeding, which suggests a similar strategy of prey acquisition in cetotheriids and early neobalaenines. Previous studies independently argued for a suction-based feeding strategy in cetotheriids, based mainly on the morphology of the mandible and the craniomandibular joint [12, 26]. No features of the cetotheriid hyoid apparatus [20, 26] are correlated with suction feeding, but a strengthened articulation of the latter with the basicranium could plausibly have played a role. Support for this idea may come from *Eschrichtius robustus*, which is the only living mysticete with an enlarged paroccipital concavity, and, concurrently, the only one known to use suction [27]. Whether *Miocaperea* itself was a suction feeder or simply retained an ancestral, suction-related morphology remains uncertain.

### Status of neobalaenines as cetotheriids

A close relationship of neobalaenines and cetotheriids or, at least, cetotheriids + balaenopteroids to the exclusion of balaenids, is indicated by a range of features preserved in *Miocaperea*. Key apomorphies mentioned above are: (i) presence of an expanded paroccipital concavity with an attendant posteroventral flange; (ii) presence of a medially projecting anteromedial corner of the pars cochlearis; (iii) presence of an anteriorly projected lateral tuberosity; and (iv) extension of the lateral lamina of the pterygoid on to the anterior process of the periotic. Further striking apomorphies include: (v) the presence of a squamosal cleft (in *Miocaperea*, *Caperea*, various cetotheriids and balaenopteroids); (vi) the presence of anteriorly pointed, keeled nasals (in *Miocaperea*, *Caperea*, *Herpetocetus* and *Piscobalaena*); and (vii) the presence of a distally expanded compound posterior process that is clearly exposed on the outer skull wall (in *Miocaperea*, *Caperea* and most cetotheriids).

Other features previously suggested to ally neobalaenines with cetotheriids and balaenopterids cannot currently be assessed for *Miocaperea*, but include characters as diverse as the anteroposterior elongation of the scapula (especially in *Caperea*, *Piscobalaena* and *Tranatocetus*) [20, 28], the loss of the first digit from the flipper (in all extant mysticetes except balaenids), the triangular shape of the coronoid process of the mandible (in *Caperea*, cetotheriids and both extinct and extant balaenopteroids) [29] and, potentially, details of the morphology of the tympanic bulla [4]. Nevertheless, the idea that neobalaenines and cetotheriids form part of a single clade has been strongly criticised by some recent studies [11, 12], primarily on two grounds: perceived dissimilarities between *Caperea* and cetotheriids (especially *Herpetocetus*), thought to preclude a close relationship [12]; and proposed similarities between *Caperea* and balaenids (together forming the Balaenoidea of some studies), thought to outweigh any resemblances of neobalaenines with cetotheriids [11]. What, if anything, can *Miocaperea* reveal about the evolution of these features in the neobalaenine lineage?

#### Perceived dissimilarities

Apparent dissimilarities cited in the literature include (i) the shape and orientation of the postglenoid process of the squamosal, described as twisted and vertically oriented in *Herpetocetus*, but transversely oriented and posteriorly reclined in *Caperea* [12]; (ii) the size and shape of the pterygoid hamulus, thought to be broadly triangular in *Herpetocetus* but small and almost indistinct in *Caperea* and balaenids [12]; (iii) the size of the pterygoid exposure on the ventral side of the skull, which is relatively small in cetotheriids, but large in *Caperea* [12]; (iv) the attachment of the anterior process of the periotic to the body of the periotic, which is strong in cetotheriids but tenuous in *Caperea* [12]; (v) the shape and location of the lateral tuberosity of the periotic, thought to be small, shelf-like and variably positioned in *Herpetocetus*, but massive and projecting far anteriorly in *Caperea* [12]; (vi) the shape of the anterior process of the periotic in medial view, described as polymorphic in *Herpetocetus*, but L-shaped in *Caperea* [12]; and (vii) the shape of the ascending process of the maxilla, described as short and parallel-sided in *Caperea*, *Balaena* and *Balaenella*, but not cetotheriids [11]. Below, each of these points is discussed in turn.

**Fig 9:**
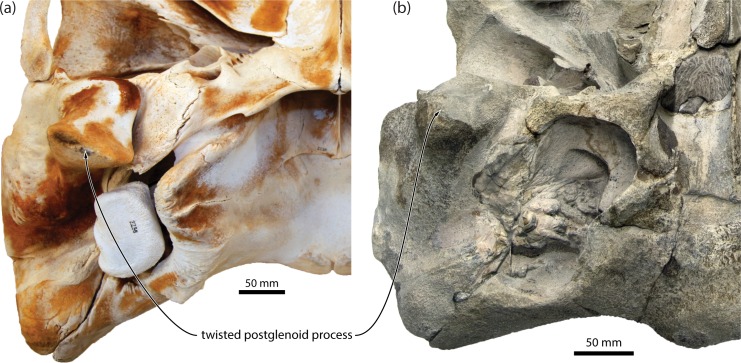
Orientation of the postglenoid process in neobalaenines. (*a*) *Caperea marginata*, NMNZ MM002235; (*b*) *Miocaperea pulchra*, SMNS 46978, both in posteroventral view.

**Fig 10:**
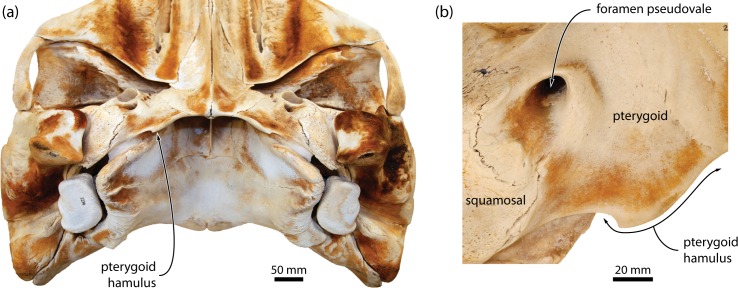
Position and shape of the pterygoid hamulus of *Caperea marginata*. (*a*) Basicranium of NMNZ MM002235 in posteroventral view; (*b*) pterygoid hamulus of the same specimen, in ventral view.

**Fig 11:**
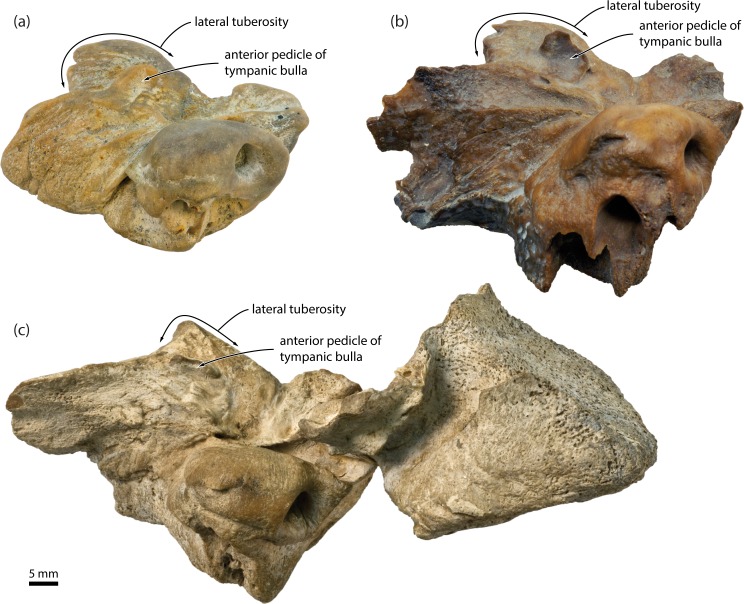
Periotic of *Herpetocetus* spp. in medial view. (*a*) *Herpetocetus morrowi*, SDNHM 63690; (*b*) *H*. *bramblei*, UCMP 82465; (*c*) *H*. *transatlanticus*, USNM 182962.

(i) The postglenoid process of *Caperea* is more transversely oriented in ventral view than that of *Herpetocetus*, but it is not perpendicular to the sagittal plane. Instead, adult individuals of *Caperea* consistently show a slight twisting of the postglenoid process in ventral view: clockwise on the left, anticlockwise on the right [4]. This twisting also occurs in somewhat more pronounced form in *Miocaperea*, where it is clearly evident despite substantial erosion of both postglenoid processes (Fig 9). Though still not as marked as in *Herpetocetus*, the orientation of the postglenoid process in *Miocaperea* is therefore consistent with a twisted ancestral postglenoid morphology. We agree that the articular surface in *Caperea* is more inclined than in *Herpetocetus* in lateral view, but note that the degree of inclination is relatively slight and exaggerated by the natural anterior slant of the *Caperea* skull (as judged from the orientation of the orbit) when resting on a horizontal surface. This is in stark contrast to balaenids, in which the articular surface is markedly more horizontal in lateral view. Erosion of the articular surface and uncertainty about the *in vivo* orientation of the skull in *Miocaperea* (owing to the apparent posterior orientation of the orbits in lateral view) currently hinder a confident assessment of the slope of the postglenoid process in this species.

(ii) *Caperea* and balaenids strikingly differ from most other mysticetes in the shape of the pterygoid hamulus. Thus, instead of being finger-like, the hamulus of *Caperea* is developed as a small, triangular, somewhat hook-shaped projection (Fig 10); by contrast, that of balaenids is expanded laterally into a broad, robust horizontal blade [24]. Given these differences, we disagree that the condition of the hamulus in *Caperea* and balaenids represents a shared state, but note that *Miocaperea* does not preserve the hamuli, thus leaving open the question of character homology. It is interesting to note that *Herpetocetus* also shows a relatively unusual morphology of the hamulus, with the latter being triangular and somewhat more confluent with the remainder of the pterygoid than in other mysticetes [12]. This loss of “distinctiveness” could plausibly be interpreted as a first step towards a state similar to *Caperea*.

(iii) *Caperea* is unusual in having an extremely large ventral exposure of the pterygoid, with the latter–uniquely among mysticetes–entirely surrounding the foramen pseudovale [19]. In this arrangement, *Caperea* differs from all other described mysticetes (including *Miocaperea*), in which the foramen pseudovale instead appears to be at least partially surrounded by the squamosal [19]. The extremely enlarged exposure of the pterygoid in *Caperea* therefore represents a phylogenetically uninformative autapomorphy.
(iv) Like the ventral exposure of the pterygoid, the narrow connection between the anterior process and the body of the periotic in *Caperea* [12] is autapomorphic, and thus phylogenetically uninformative. *Miocaperea* instead shows the widespread plesiomorphic condition of a solidly attached anterior process (Fig 1).
(v) The position of the lateral tuberosity in cetotheriids is variable, both inter- and intraspecifically, which has led to the phylogenetic value of this feature being questioned [12]. This is particularly true for *Herpetocetus*, in which the lateral tuberosity can occur posterolateral, lateral or anterolateral to the anterior pedicle of the tympanic bulla, depending on the specimen and species. Nevertheless, it appears that one of the extremes of this continuum–the plesiomorphic position of the tuberosity posterolateral to the anterior pedicle–only occurs in extremely juvenile individuals (e.g. SDNHM 38689), suggesting that change in position may be ontogenetic [12]. Most *Herpetocetus* specimens instead have a tuberosity that is located lateral (e.g. *H*. *morrowi*: SDNHM 34155; *H*. *transatlanticus*: USNM 182962) or anterolateral (e.g. *H*. *bramblei*: UCMP 82465; *H*. *morrowi*: SDNHM 63690; *Herpetocetus* sp.: NMNS PV19540) to the anterior pedicle (Fig 11). In *Caperea*, the lateral tuberosity is developed as a broad, relatively massive shelf extending all along the anterior process, well past the level of the anterior pedicle [4]. By contrast, *Miocaperea* preserves a more *Herpetocetus*-like state, in which the lateral tuberosity is located further posteriorly and approximately lateral to the anterior pedicle (Fig 1).

The question of whether the shape of the lateral tuberosity is comparable between neobalaenines and *Herpetocetus* is more problematic. The lateral tuberosity of *Herpetocetus* is relatively variable in both size and shape, ranging from small and triangular (e.g. *H*. *transatlanticus*: USNM 182962) to proportionally large and rounded (e.g. *H*. *bramblei*: UCMP 82465; *Herpetocetus* sp.: NMNS PV19540). In all cases, however, the tuberosity is bent laterally, articulates with the adjacent squamosal and, in general, is oriented anterolaterally in ventral view, relative to the long axis of the anterior process (Fig 11). The lateral tuberosity of *Caperea* provides the closest match for that of *Herpetocetus* in also being oriented anterolaterally and in articulating with the squamosal [4], yet at the same time clearly differs in being considerably more massive. *Miocaperea* currently offers little to clarify this situation–partly, because the anterior face of the lateral tuberosity remains covered in matrix, and partly because the rim of the squamosal surrounding the anterior process is crushed.

(vi) The shape of the anterior process is demonstrably variable among cetotheriids: two-bladed, or L-shaped, in *Kurdalagonus mchedlidzei*, *Herpetocetus transatlanticus*, *H*. *bramblei* and, possibly, *Brandtocetus chongulek* (Fig 11); and triangular or squared in *Piscobalaena nana*, *H*. *morrowi* and *Metopocetus durinasus*. In *Caperea*, the anterior process is also L-shaped [4]. In *Miocaperea*, the outline of the anterior process is partially obscured by the lateral lamina of the pterygoid; it appears, however, that the anterior border of the process, if not L-shaped, is at least concave (Fig 1). Such polymorphism can be a challenge to phylogenetics, but does not in itself invalidate a particular character. In this case, the outline of the anterior process is admittedly not clear-cut, but we note that an irregularly-shaped anterior process is relatively rare among mysticetes, and currently confined to cetotheriids, neobalaenines and eomysticetids [6, 30].

**Fig 12:**
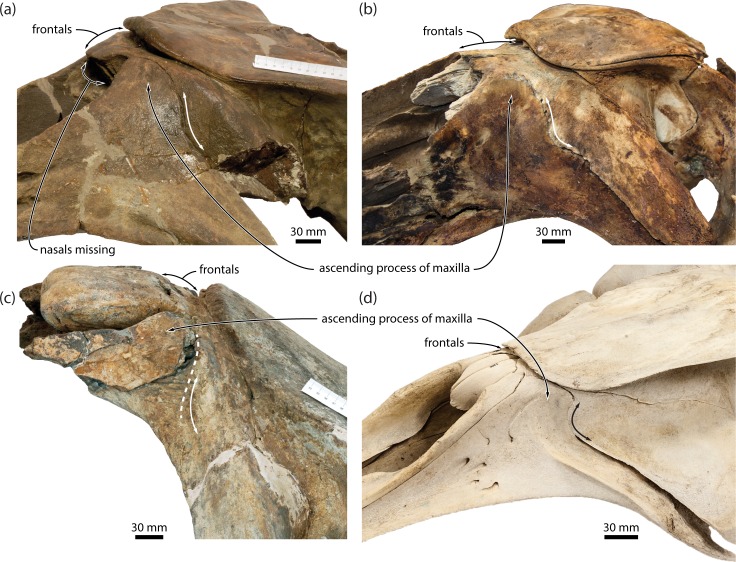
Ascending process of the maxilla in balaenids and *Caperea*. (*a*) *Balaenella brachyrhynus*, NMB 42001, in anterolateral view; (*b*) neonate of *Balaena mysticetus*, LACM 54485, in anterolateral view; (*c*) *Balaenula astensis*, MSNTUP I12555, in lateral view; (*d*) juvenile of *Caperea marginata*, NMNZ MM002254, in anterolateral view. Note the missing nasals of *B*. *brachyrhynus*, as well as the relatively obtuse posterior angle defining the ascending process of balaenids.

One previous study questioned the homology of the L-shaped anterior process in *Caperea* and cetotheriids, pointing out that the irregular anterior border in *Caperea* is formed entirely within the process, whereas in *H*. *transatlanticus* it arises from an interaction of the anterior process and the pars cochlearis [12]. This distinction is, however, somewhat arbitrary, and in itself variable. Thus, the L-shape is clearly formed entirely within the anterior process in *K*. *mchedlidzei* (NMRA 10476/1), *B*. *chongulek* (TNU skull 2), *H*. *bramblei* (UCMP 82465; Fig 11) and a periotic of *Herpetocetus* sp. from the Lee Creek Mine, North Carolina, USA (USNM 360765). The condition in the holotypes of *H*. *transatlanticus* (USNM 182962) and *Miocaperea* is less clear, but largely depends on where one draws the line between the pars cochlearis and the anterior process. In our view, it is entirely reasonable to argue that the L-shape, or concavity, in both is also entirely formed by the anterior process.

(vii) The shape of the ascending process of the maxilla in adult *Caperea* and balaenids is difficult to determine because its size is restricted by the anterior telescoping of the supraoccipital (see below). Nevertheless, a clearly parallel-sided ascending process is evident in several neonate (NMNZ MM002262, MM002898) and juvenile *Caperea* (NMNZ MM002254; Fig 12), as well as *Miocaperea*, which–irrespective of the length of the process–therefore seem to share this condition with most cetotheriids and balaenopterids [29]. A parallel-sided ascending process of the maxilla also occurs in some individuals of *Balaena mysticetus* [11] (e.g. LACM 54464), but in other specimens, including neonates (e.g. LACM 54485), it is much more triangular (Fig 12). Other balaenids, including *Balaenula astensis* (MSNTUP I12555), neonate specimens of *Eubalaena* spp. (e.g. CNPMAMM 746 and LACM 54763), *Morenocetus parvus* [29] and, contrary to previous claims [11], *Balaenella brachyrhynus* (NMB 42001), also have a triangular or rounded ascending process with posteriorly convergent medial and lateral borders, and thus clearly differ from balaenopterids, most cetotheriids and neobalaenines in this regard (Fig 12).

#### Proposed balaenoid synapomorphies

In terms of proposed similarities between *Caperea* and balaenids, one recent study listed 15 morphological features which it argued were shared by both, and concluded that the weight of the available evidence therefore spoke against a relationship of *Caperea* with cetotheriids [11]. Specifically, these features included:

“(1) massive elongation of supraoccipital; (2) supraoccipital covering the parietal and excluding the parietal to be exposed in dorsal view; (3) anterior end of supraoccipital covering the posterior portion of the interorbital region of the frontal; (4) parietal not extending anteriorly to the posteromedial elements of the rostrum; (5) short ascending process of the maxilla that may be squared in some individuals; (6) premaxilla evident laterally to the nasal; (7) lack of parietal exposure at cranial vertex; (8) development of a concave posterior wall of the temporal fossa; (9) zygomatic process of the squamosal strongly shortened; (10) low tympanic cavity; (11) low conical process of the tympanic bulla; (12) dorsoventrally oriented mandibular condyle; (13) presence of a depression or a groove for mylohyoidal muscle on the medial side of the dentary; (14) fusion of cervical vertebrae; and (15) long baleen.” [11: 15]

**Fig 13:**
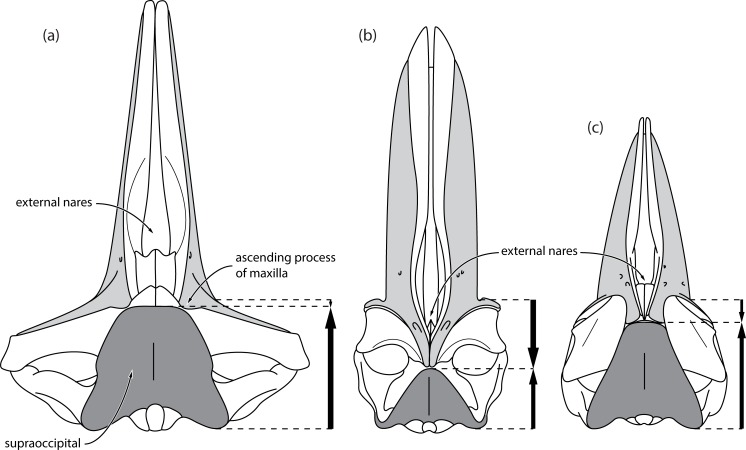
Reciprocal relationship between the ascending process of the maxilla and the supraoccipital shield. (*a*) *Eubalaena*; (*b*) *Piscobalaena*; (*c*) *Balaenoptera*, all in dorsal view. All skulls are scaled so that the anteroposterior distance between the antorbital notch and the posterior border of the exoccipital is the same. Arrows indicate the dominant mode of telescoping. The anterior telescoping of the supraoccipital (dark grey) is accompanied by a shortening of the ascending processes of the maxillae (light grey), and *vice versa*.

Of these 15 purported balaenoid synapomorphies, seven directly or indirectly describe the same feature, namely, the anterior extension of the supraoccipital shield. Thus, as the supraoccipital extends anteriorly (**1**), it excludes the parietal both from the vertex (**7**) and from dorsal view (**2**), and covers the interorbital region of the frontal (**3**). As it extends forward, the supraoccipital leaves no room for the rostral bones (maxilla, premaxilla and nasal) to project posteriorly on to the vertex, thus precluding overlap of the rostral bones with the parietal (**4**) and resulting in a short ascending process of the maxilla (**5**), with the latter–like the nasal–also being unable to extend posterior to the premaxilla (**6**). All seven of these characters (in particular chars **1–5** and **7**) are therefore linked by a simple, reciprocal geometrical relationship: the more the supraoccipital extends anteriorly, the less room there is for the rostral bones to telescope backwards, and *vice versa* (Fig 13). Functionally, this relationship is presumably constrained by the relatively short intertemporal region of crown mysticetes, which reduces the space available for telescoping; the need to maintain enough of an attachment surface for the semispinalis capitis muscle to support the skull; and the need to position the external nares as far posteriorly and/or dorsally as possible to facilitate breathing.

Balaenids, neobalaenines and cetotheriids provide perfect case studies: in the former two, the supraoccipital occupies much of the intertemporal portion of the cranium at the expense of the rostral bones; by contrast, exactly the opposite is true in cetotheriids, where the pronounced posterior elongation of the rostral bones confines the supraoccipital shield to the posteriormost portion of the cranium (Fig 13). Note, however, that in balaenids the ascending process of the maxilla may be genuinely (i.e. ancestrally) short, as judged from the often relatively large exposure of the frontal on the vertex (e.g. in *Balaenella brachyrhynus* and neonate *Eubalaena glacialis*, LACM 54763). This condition is especially obvious in the oldest described balaenid, *Morenocetus parvus* [31], thus making it the only right whale in which the ascending process of the maxilla can be coded without its relative position being obviously compromised by the supraoccipital. In extant balaenopterids, the anterior telescoping of the supraoccipital and the concurrent posterior shift of the rostral bones are more balanced, resulting in an anteriorly truncated supraoccipital shield that meets the equally truncated, squared ascending processes of the maxillae roughly halfway along the vertex.

**Fig 14:**
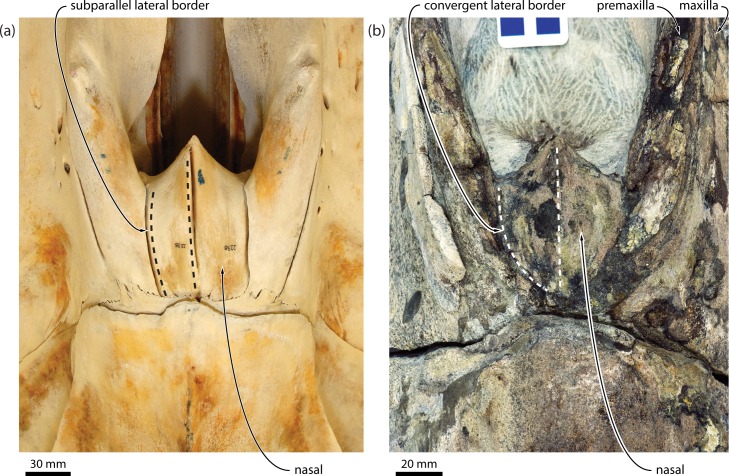
Detailed view of the vertex showing the dorsal outline of the nasals. (*a*) *Caperea marginata*, NMNZ MM002235; (*b*) *Miocaperea pulchra*, SMNS 46978. Note the subparallel lateral and medial margins of the nasal in *C*. *marginata*, compared to posteriorly convergent margins in *M*. *pulchra*.

Overall, the above examples demonstrate that the first seven features cited in support of grouping *Caperea* with balaenids are interdependent, and thus–for taxa showing pronounced telescoping–should be coded only once to avoid incidental character weighting. In the present analysis, all of these features are therefore subsumed in char. 90, “Anteriormost point of supraoccipital in dorsal view”, which we accept as a potential balaenoid synapomorphy. Consider, however, that neobalaenines differ from balaenids in the detailed arrangement of their skull vertex: whereas the frontal has virtually disappeared from view behind the nasals in both *Caperea* and *Miocaperea* (Fig 14), it has escaped obliteration by the supraoccipital in balaenids by insertion between the posterior portions of the rostral bones (Fig 12) (pl. 42 in [32]). This difference in vertex architecture suggests that the pronounced telescoping of the supraoccipital and attendant changes in balaenids and neobalaenines are a result of evolutionary convergence. *Miocaperea* further differs in having posteriorly convergent nasals, accompanied by equally convergent ascending processes of the maxilla and premaxilla (Fig 14). By contrast, the lateral margins of the nasals in balaenids, and indeed *Caperea*, are nearly parallel (unclear in the holotype of *Balaenella brachyrhynus*, in which the nasals are missing, contra [33]; Fig 12). This condition is consistent with the common ancestor of *Caperea* and *Miocaperea* having posteriorly convergent maxillae (as seen in other cetotheriids), which were later shortened and made less convergent by the anterior telescoping of the supraoccipital.

Out of the remaining characters, we concur that a short zygomatic process of the squamosal (**9**) and long baleen (**15**) could be seen to unite *Caperea* and balaenids–assuming that the presence of long baleen is coded in lieu of the presence of an arched rostrum. Nevertheless, the case for a homologous reduction in the size of the zygomatic process is somewhat speculative, given the rather disparate morphologies of neobalaenines and balaenids in this regard (Fig S4 in [4]). In *Caperea* and, as far as can be told, *Miocaperea*, the zygomatic process is reduced to the point of obliteration, but seems to be oriented anteriorly judging from the position of the (usually juxtaposed) postorbital process of the frontal. By contrast, the zygomatic process of balaenids is typically much better developed, twisted in anterior view, and oriented anterolaterally. Further evidence is needed to demonstrate that these conditions are plausibly homologous.

**Fig 15:**
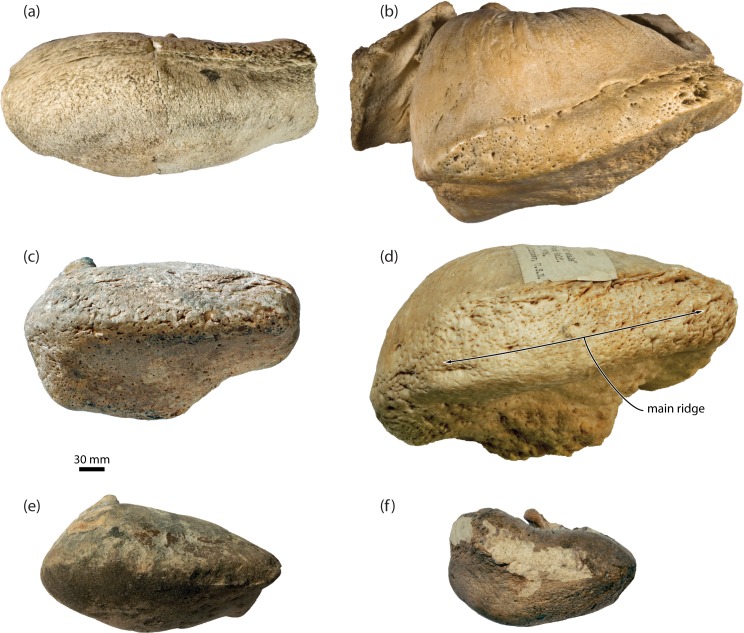
Comparison of the tympanic bulla of *Caperea*, balaenids and *Herpetocetus* in medial view. (*a*) *Caperea marginata*, OM VT227; (*b*) *Eubalaena australis*, NMNZ MM000226; (*c*) *Balaenula astensis*, MSNTUP I12555; (*d*) *Balaena mysticetus*, USNM 15695; (*e*) *Balaenella brachyrhynus*, NMB 42001; (*f*) *Herpetocetus transatlanticus*, USNM 182962. *E*. *australis* and *B*. *astensis* have been mirrored to facilitate comparisons.

It is unclear how to interpret the statements that *Caperea* and balaenids share (**8**) a “concave posterior wall of the temporal fossa” (p. 15 in [11]), as the direction of the concavity was not specified. Confusingly, the corresponding character (number 76, “Strong concavity in temporal fossa posterior to emergence of supraorbital process of frontal”; p. 10 of the suppl. material of [11]) appears to be coded as “present” (state 1) for *Caperea*, *Zygorhiza kochii*, *Aetiocetus weltoni* and balaenopterids (including grey whales), but not a single balaenid, thus invalidating this character as a potential balaenoid synapomorphy. Likewise, we are unsure about the correct interpretation of the presence of a “low tympanic cavity” (**10**). We agree that the tympanic bulla of *Caperea* is dorsoventrally flattened in medial view, but the same cannot necessarily be said for balaenids, in which this compression appears to be limited to the main ridge (Fig 15). *Peripolocetus vexillifer* is an exception, but even in this case the dorsoventral compression of the bulla appears less marked than in *Caperea*. Additional data on the bulla morphology of *Miocaperea* and archaic balaenids, such as *Morenocetus* and *Peripolocetus*, are necessary to test whether a flattened bulla might indeed represent a shared feature.

**Fig 16:**
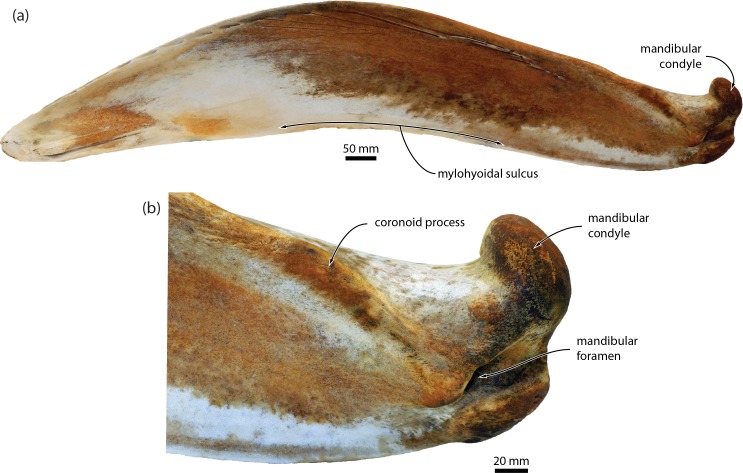
Mandible of *Caperea marginata* in medial view. (*a*) NMNZ MM002235; (*b*) detailed view of the posterior (ramus) portion of the same specimen.

We disagree with the claim that the presence of a low conical process of the tympanic bulla is demonstrably homologous in balaenids and *Caperea*, but not *Herpetocetus* (**11**) [11]. All three of these taxa appear to show a comparable degree of reduction and dorsal flattening of the conical process, and thus do not allow an *a priori* distinction into different character states. A more stringent test of homology might be whether the conical process of fossil neobalaenines is taller than in *Caperea* and, if so, whether its morphology more closely resembles that of cetotheriids or balaenids. At present, there is no material that could provide such insights. We also disagree that *Caperea* and balaenids share a dorsally oriented articular condyle of the mandible (**12**); rather, the condyle in *Caperea* appears to point posterodorsally (Fig 16), but we note that its mandible is difficult to orient (owing to the pronounced curvature of the mandibular body), and that the *in situ* position of the condyle needs to be ascertained via dissection. Finally, we agree that the presence of a mylohyoid groove or depression (**13**) and fusion of the cervical vertebrae (**14**) characterise both *Caperea* and balaenids. We also note, however, that the attachment of the mylohyoid is less clearly developed in *Caperea*, and that an apparent mylohyoid groove also occurs in at least some cetotheriids (e.g. *Herpetocetus morrowi* [12], and likely also *Piscobalaena nana*). Likewise, incipient fusion of the cervical vertebrae is present in some specimens of *Herpetocetus*, with C2 and C3 being incipiently fused in the type specimen of *H*. *morrowi* (UCMP 124950) [12], and C2–4 being partially fused in *Herpetocetus* sp. from Japan (NMNS PV-19540). Both characters could thus potentially also unite *Caperea* with (certain) cetotheriids.

In summary, only three of the 15 characters cited as balaenoid synapomorphies, namely, numbers **1**, **9** and **15**, unequivocally support a neobalaenine-balaenid clade. The remainder either code for the same feature (**2–7**), are equivocal or do not apply (**8**, **10** and **12**), or are shared by neobalaenines, balaenids *and* at least some cetotheriids (**11**, **13** and **14**). This relatively weak morphological support for Balaenoidea is outweighed by the morphological evidence supporting a neobalaenine-cetotheriid clade, as well as the fact that molecular analyses consistently group *Caperea* with extant balaenopteroids [8, 9]. As a result, we therefore here strongly reaffirm our previous referral of neobalaenines to Cetotheriidae [4], and our identification of *Caperea* as the last survivor of this once diverse family.

## Supporting Information

S1 Fig Right periotic of *Miocaperea pulchra* (SMNS 46978), in ventral view. Note the position and ventral flooring of the infilled facial sulcus.
(TIF)

S2 Fig Full results of the total evidence phylogenetic analysis. Majority-rule consensus tree showing all compatible clades (“allcompat” option in MrBayes). Numbers next to branches are posterior probabilities, with only values ≥ 50% shown.
(TIF)

S1 File Nexus file containing the full total evidence data matrix. (NEX)

## Abbreviations

LACM: Natural History Museum of Los Angeles County, Los Angeles, USA
MNHN: Museum National d`Histoire Naturelle, Paris, France
MSNTUP: Museo di Storia Naturale e del Territorio, Università di Pisa, Pisa, Italy
NMB: Natuurmuseum Brabant, Tilburg, The Netherlands
NMNS: National Museum of Nature and Science, Tokyo/Tsukuba, Japan
NMNZ: Museum of New Zealand Te Papa Tongarewa, Wellington, New Zealand
NMRA: National Museum of the Republic of Adygeya, Maikop, Russia
OM: Otago Museum, Dunedin, New Zealand
OU: Geology Museum, University of Otago, Dunedin, New Zealand
SDNHM: San Diego Natural History Museum, San Diego, USA
SMNS: Staatliches Museum für Naturkunde, Stuttgart, Germany
TNU: Department of Zoology, Taurida National University, Simferopol, Ukraine
UCMP: University of California Museum of Paleontology, Berkeley, USA
USNM: National Museum of Natural History, Smithsonian Institution, Washington DC, USA
ZMT: Fossil mammals catalogue, Canterbury Museum, Christchurch, New Zealand.
