# Supplementary figures and images for: A Link No Longer Missing: New Evidence for the Cetotheriid Affinities of Caperea
Source: PLoS One. 2016 Oct 6;11(10):e0164059. doi: 10.1371/journal.pone.0164059 (PMC5053404; doi:10.1371/journal.pone.0164059)

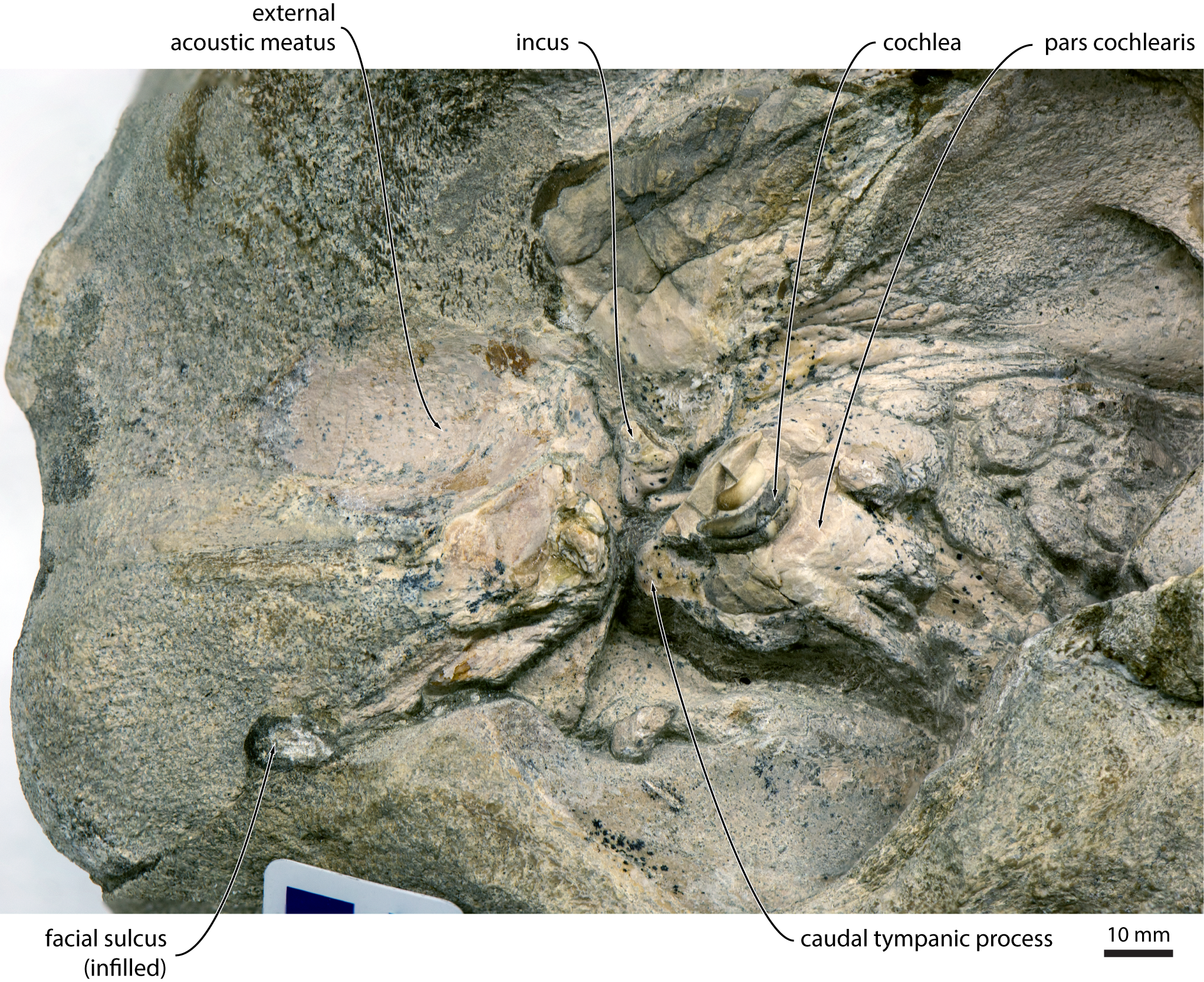

Supplement: S1 Fig — Note the position and ventral flooring of the infilled facial sulcus. (TIF) [file pone.0164059.s001.tif]

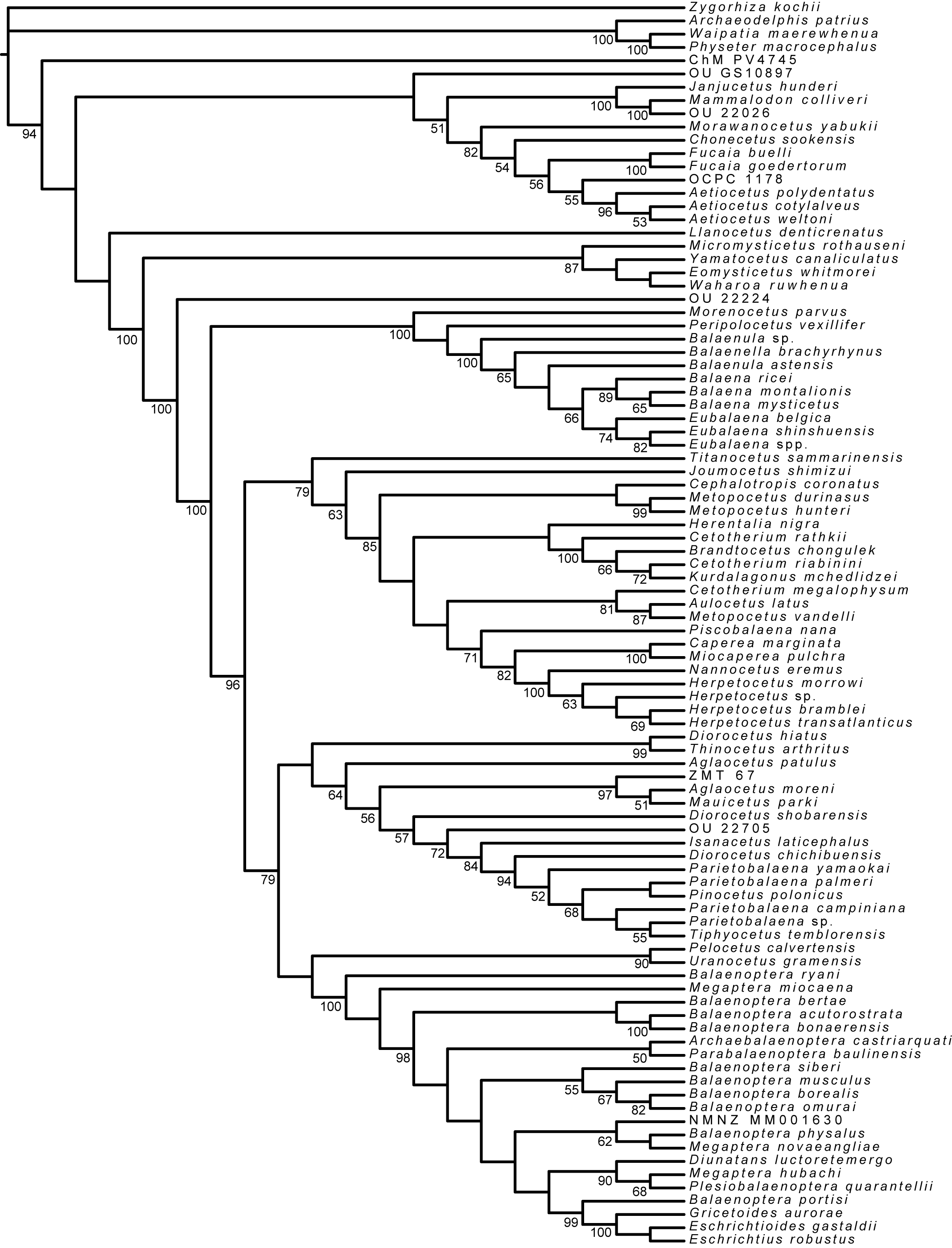

Supplement: S2 Fig — Majority-rule consensus tree showing all compatible clades (“allcompat” option in MrBayes). Numbers next to branches are posterior probabilities, with only values ≥ 50% shown. (TIF) [file pone.0164059.s002.tif]
